# Supplementary material for: Enhanced Efficacy of Vaccination With Vaccinia Virus in Old vs. Young Mice
Source: Front Immunol. 2019 Jul 31;10:1780. doi: 10.3389/fimmu.2019.01780 (PMC6685358; doi:10.3389/fimmu.2019.01780)
Supplement: Supplementary file 1 [file Data_Sheet_1.pdf]

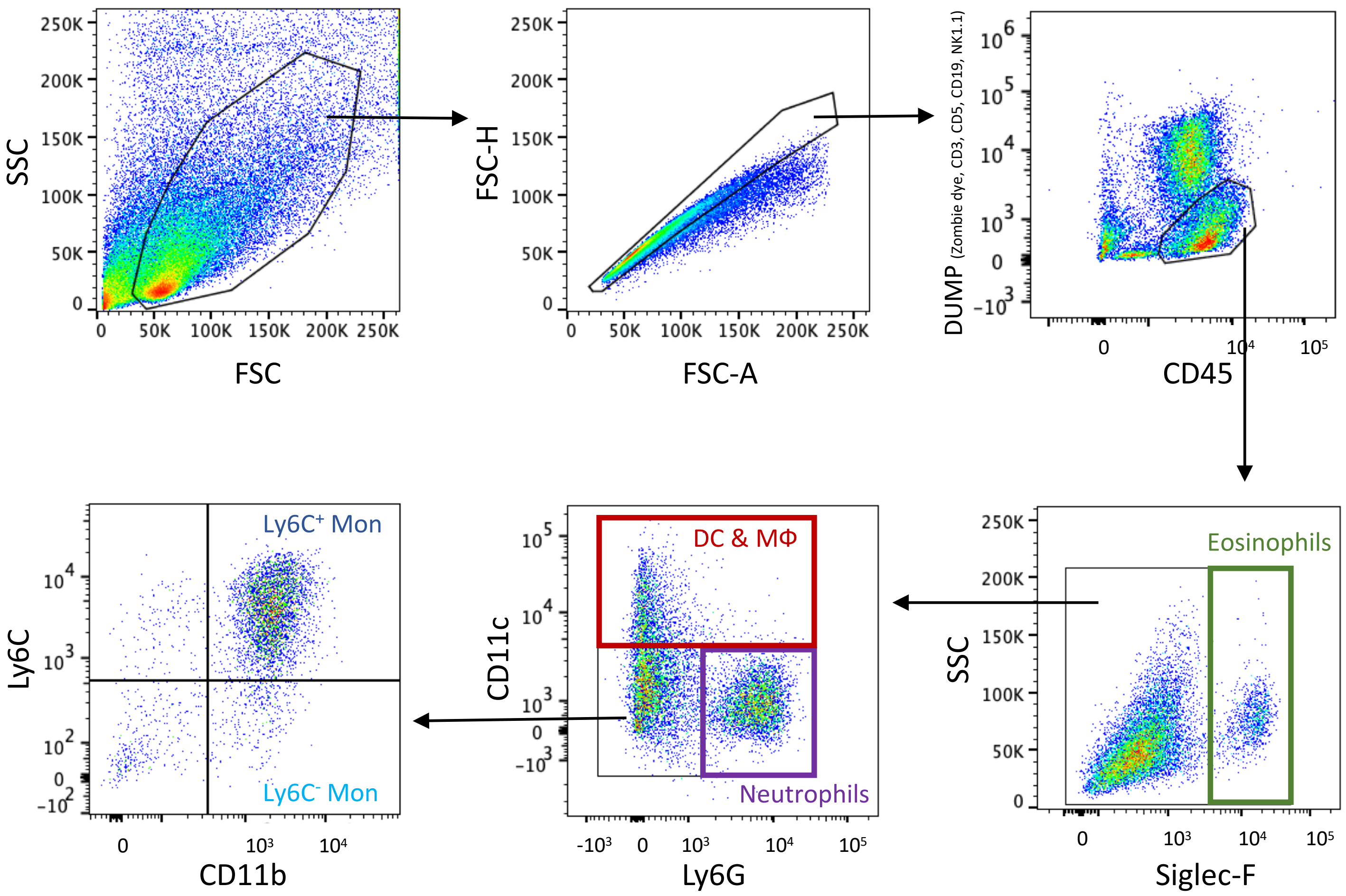

Supplementary figure 1. Gating strategy for flow cytometry of myeloid lineage cells present in mouse ear tissue 7 d after intradermal injection with 10^4 PFU of VACV strain WR. Cells were gated on their ability to scatter light. Doublets were excluded using a FSC-A versus FSC-H plot. The myeloid gate included $CD45^+$Zombie $dye^-CD3^-CD5^-CD19^-NK1.1^-$ cells. Further myeloid cell subpopulations were classified as follows: Eosinophils: $CD45^+CD3^-CD5^-CD19^-NK1.1^-CD11c^-$ Siglec- F^+; DC and MΦ (dendritic cells and macrophages): $CD45^+CD3^-CD5^-CD19^-NK1.1^-$Siglec-$F^-$ $CD11c^+$; Neutrophils: $CD45^+CD3^-CD5^-CD19^-NK1.1^-CD11c^-$Siglec-$F^-$ $Ly6G^+$; $Ly6C^+$Mon (inflammatory monocytes): $CD45^+CD3^-CD5^-CD19^-NK1.1^-CD11c^-$Siglec-$F^-Ly6G^-CD11b^+Ly6C^+$; $Ly6C^-$Mon (residential monocytes): $CD45^+CD3^-CD5^-CD19^-NK1.1^-CD11c^-$Siglec-$F^-Ly6G^-$ $CD11b^+Ly6C^-$.

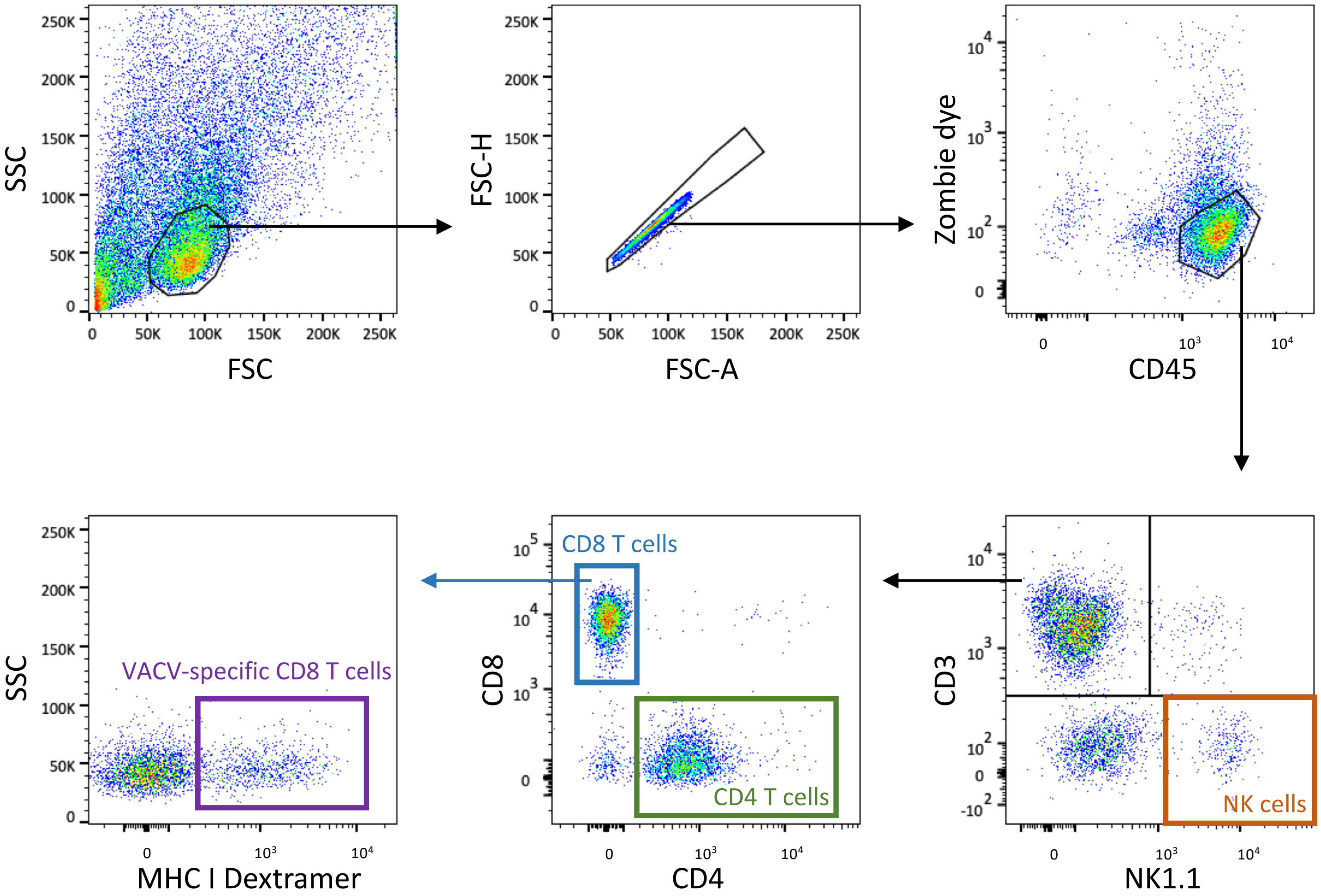

Supplementary figure 2. Gating strategy for flow cytometry of lymphoid lineage cells present in mouse ear tissue 7 d after intradermal injection with 10^4 PFU of VACV strain WR. Cells were gated on their ability to scatter light. Doublets were excluded using a FSC-A versus FSC-H plot. Then, hemopoietic cells were gated as $CD45^+$Zombie dye^- cells. Further lymphoid subpopulations were classified as follows: NK cells: $CD45^+CD3^-NK1.1^+$; CD4 T cells: $CD45^+CD3^+NK1.1^-CD8^-CD4^+$; CD8 T cells: $CD45^+CD3^+NK1.1^-CD4^-CD8^+$; VACV-specific CD8 T cells: $CD45^+CD3^+NK1.1^-CD4^-CD8^+Dextramer^+$.

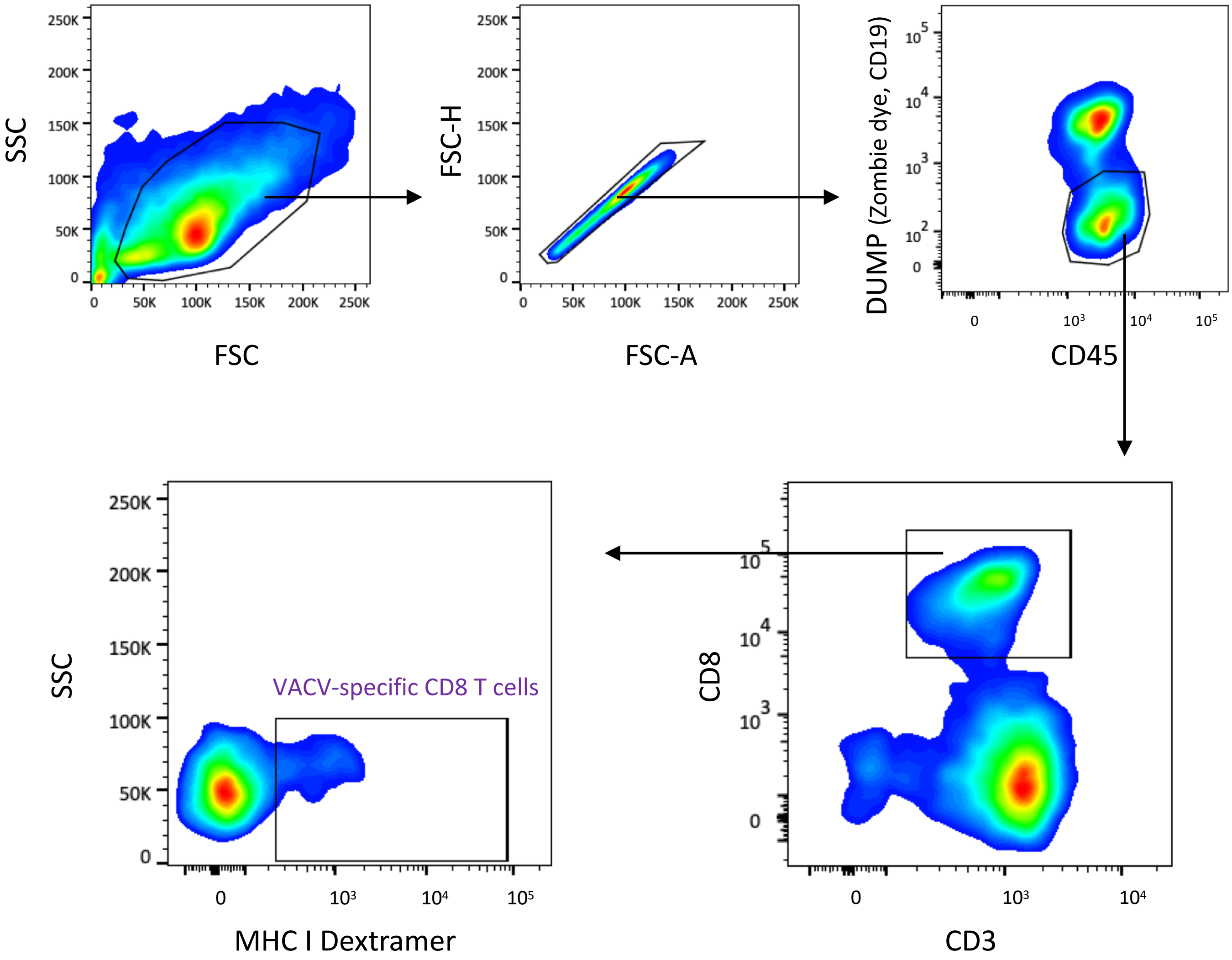

Supplementary figure 3. Gating strategy for flow cytometry of VACV-specific CD8 T cells obtained from draining cervical lymph nodes 7 d post intradermal injection of mouse ear pinnae with 10^4 PFU of VACV strain WR. Cells were gated on their ability to scatter light, and doublets were excluded using a FSC-A versus FSC-H plot. Then, VACV-specific CD8 T lymphocytes were gated as $CD45^+CD3^+NK1.1^-CD4^-CD8^+Dextramer^+$cells.

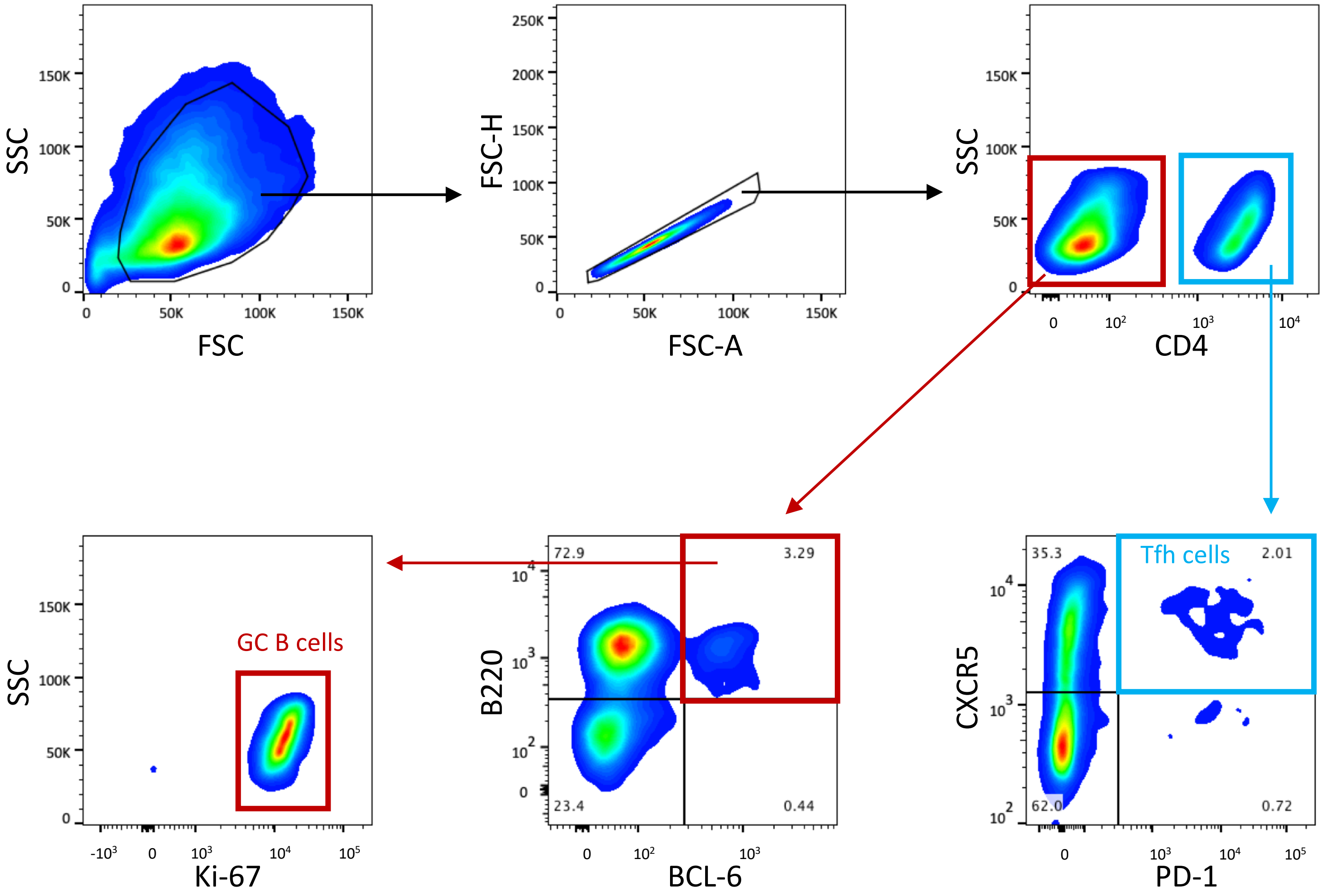

Supplementary figure 4. Gating strategy for flow cytometry of germinal centre (GC) B cells and T follicular helper (Tfh) lymphocytes from draining cervical lymph nodes 7 d post intradermal infection of mouse ear pinnae with 10^4 PFU of VACV strain WR. Cells were gated on their ability to scatter light. Doublets were excluded using a FSC-A versus FSC-H plot. Further lymphoid subpopulations were classified as follows: GC B cells: $CD4^-B220^+BCL\text{-}6^+Ki\text{-}67^+$; Tfh cells: $CD4^+CXCR5^+PD\text{-}1^+$.

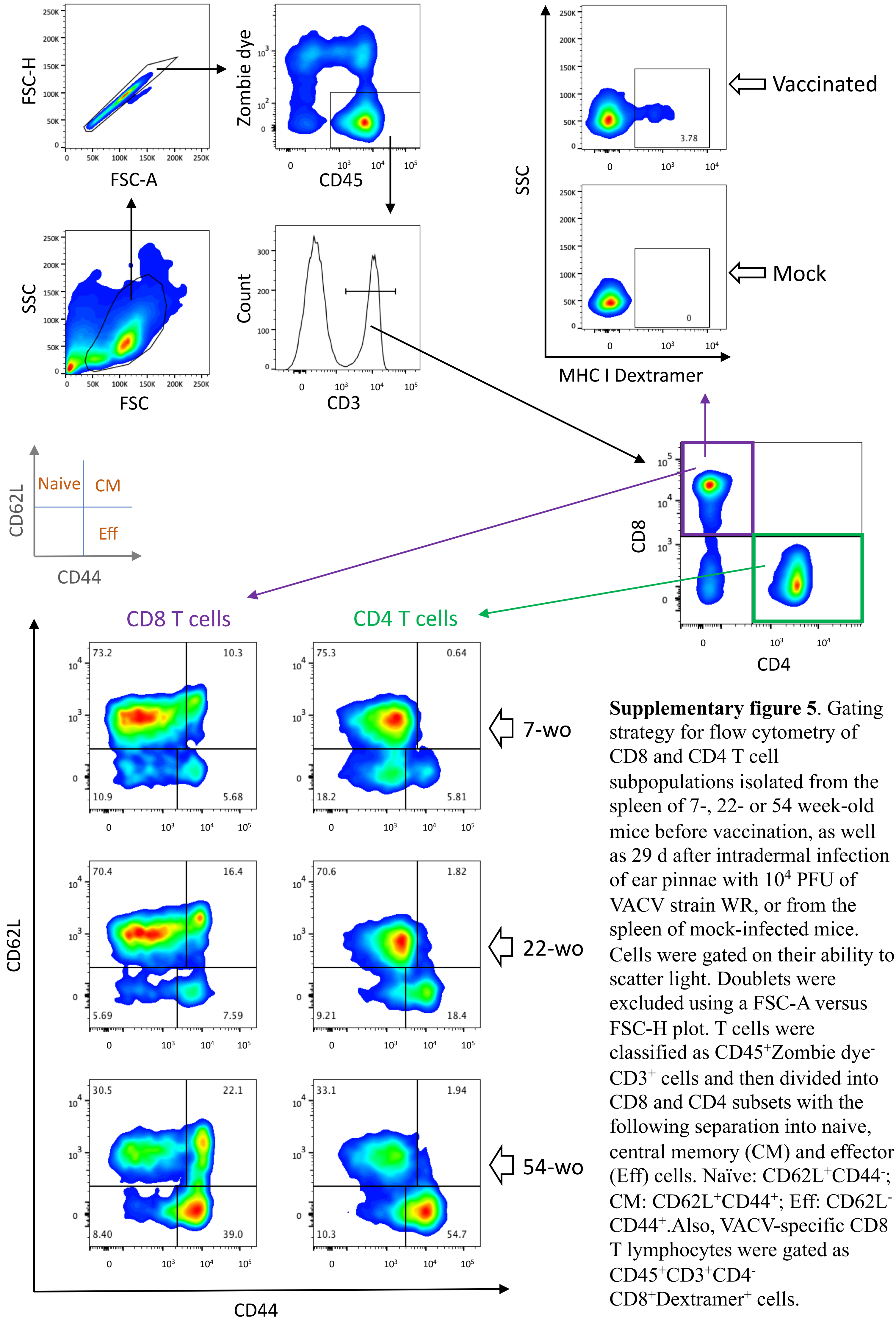

Supplementary figure 5. Gating strategy for flow cytometry of CD8 and CD4 T cell subpopulations isolated from the spleen of 7-, 22- or 54 week-old mice before vaccination, as well as 29 d after intradermal infection of ear pinnae with 10^4 PFU of VACV strain WR, or from the spleen of mock-infected mice. Cells were gated on their ability to scatter light. Doublets were excluded using a FSC-A versus FSC-H plot. T cells were classified as $CD45^+$Zombie dye^- $CD3^+$ cells and then divided into CD8 and CD4 subsets with the following separation into naive, central memory (CM) and effector (Eff) cells. Naïve: $CD62L^+CD44^-$; CM: $CD62L^+CD44^+$; Eff: $CD62L^-$ $CD44^+$.Also, VACV-specific CD8 T lymphocytes were gated as $CD45^+CD3^+CD4^-$ $CD8^+$$Dextramer^+$ cells.

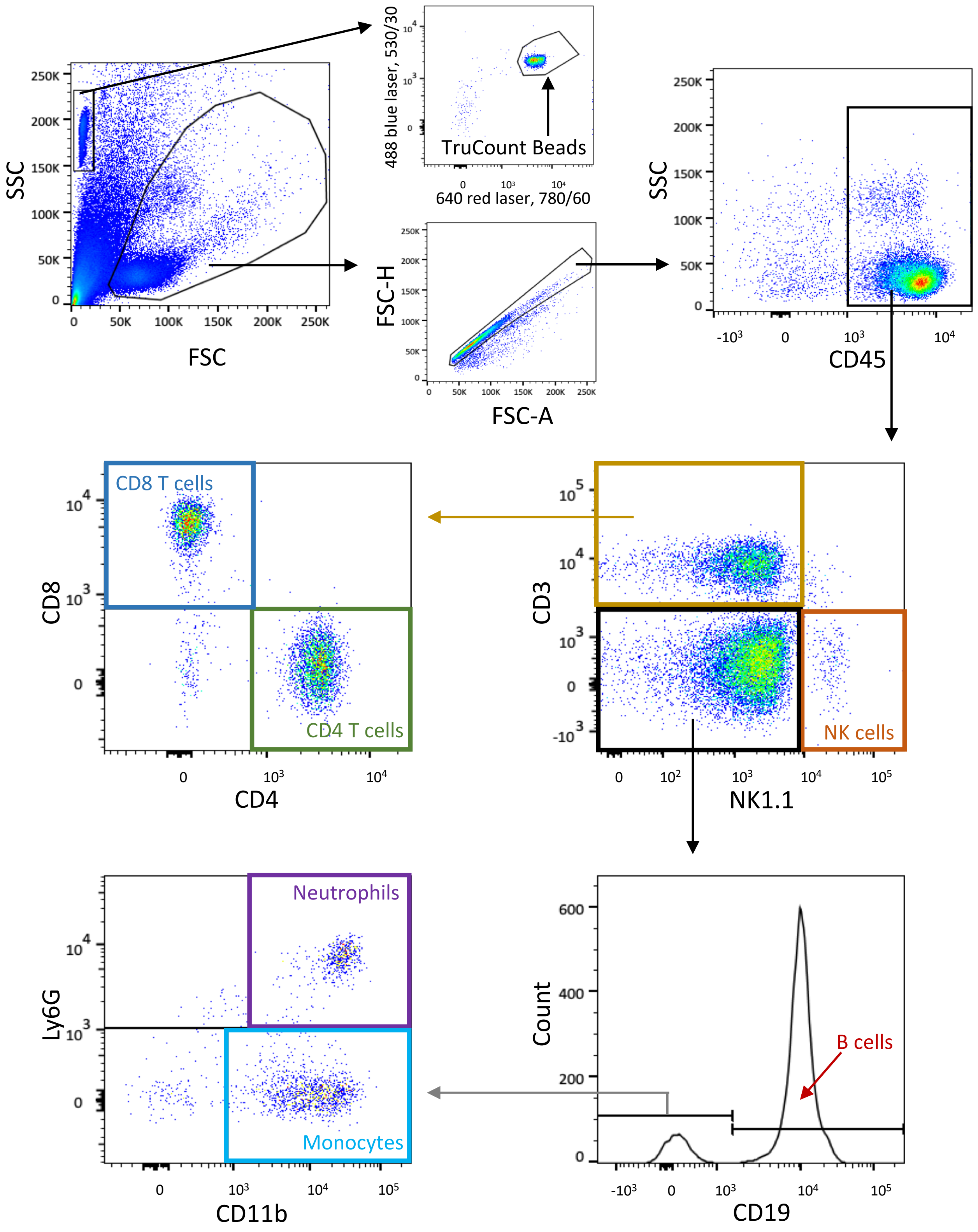

Supplementary figure 6. Gating strategy for Trucount flow cytometry of different leukocyte populations present in mouse blood. First, cell and bead areas were identified based on their ability to scatter light. Then the beads were gated based on their bright fluorescence in a variety of channels. Cell doublets were excluded using a FSC-A versus FSC-H plot, then hemopoietic cells were gated as CD45+cells. Further leukocyte subpopulations were classified as follows: NK: $CD45^{+}CD3^{-}NK1.1^{+}$; CD4 T cells: $CD45^{+}CD3^{+}NK1.1^{-}CD8^{-}CD4^{+}$; CD8 T cells: $CD45^{+}CD3^{+}NK1.1^{-}CD4^{-}CD8^{+}$; B cells: $CD45^{+}CD3^{-}NK1.1^{-}CD19^{+}$; Neutrophils: $CD45^{+}CD3^{-}NK1.1^{-}CD19^{-}CD11b^{+}Ly6G^{+}$; Monocytes: $CD45^{+}CD3^{-}NK1.1^{-}CD19^{-}CD11b^{+}Ly6G^{-}$.

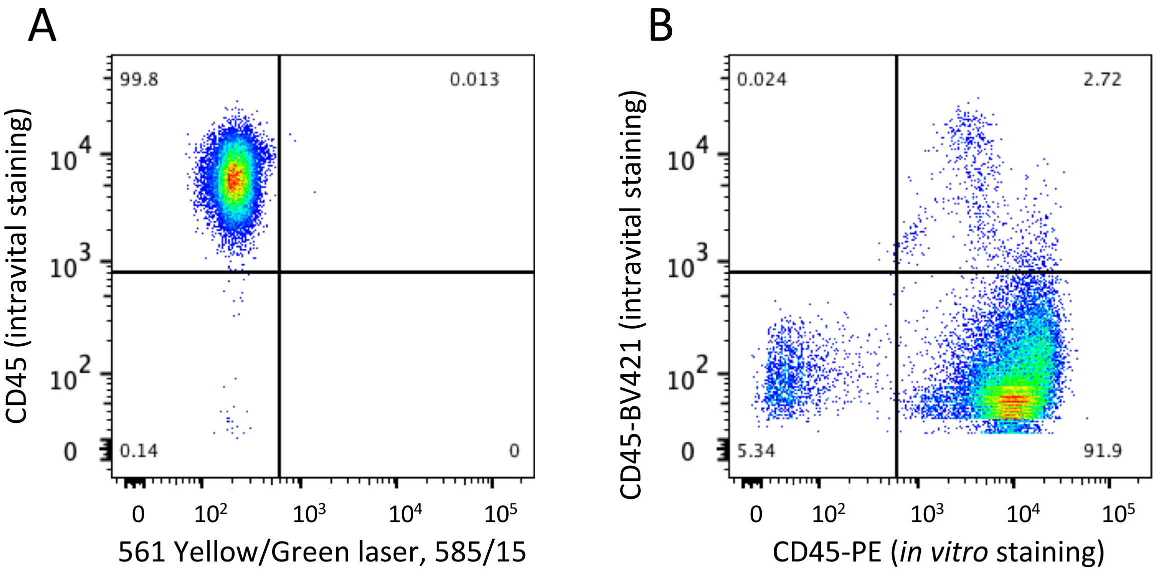

Supplementary figure 7. Intravascular and *in vitro* staining with anti-CD45 antibodies. (**A**) Flow cytometry of blood sample after intravascular staining with anti-CD45 antibodies. (**B**) Flow cytometry of cells from the ear of a mouse 7 d after intradermal infection with 10^4 PFU of VACV strain WR after intravascular staining with anti-CD45-BV421 antibodies followed by *in vitro* staining with anti-CD45-PE antibodies.

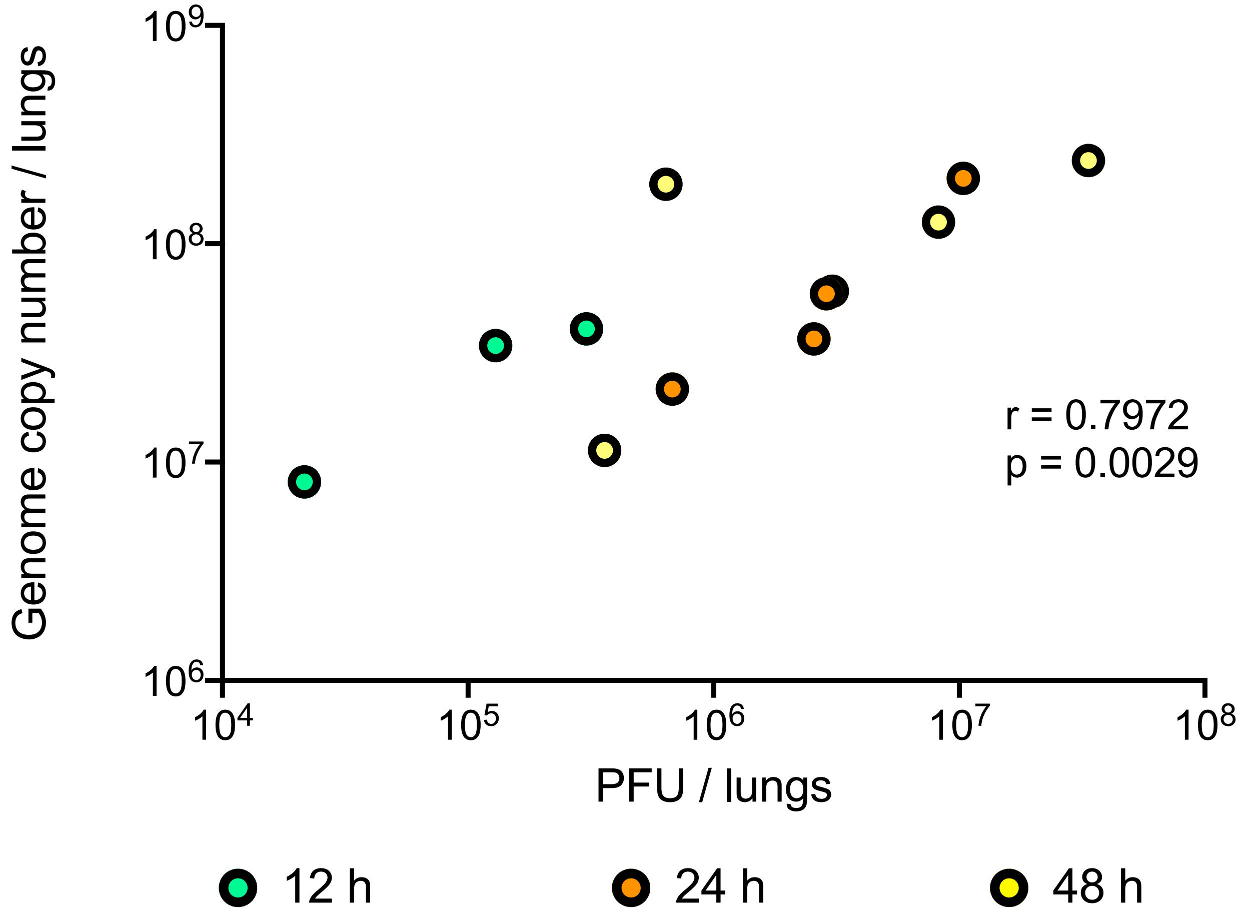

Supplementary figure 8. Spearman correlation analysis of infectious virus titer versus virus genome copy number. VACV loads were measured in the lungs of naïve mice at 12, 24, or 48 h after intranasal infection with 0.7×10^7 PFU of VACV WR by either plaque assay on BSC-1 cells (expressed as plaque-forming units [PFU]/ lungs), or by qPCR using primers for the VACV *E9L* gene (expressed as genome copy number / lungs).

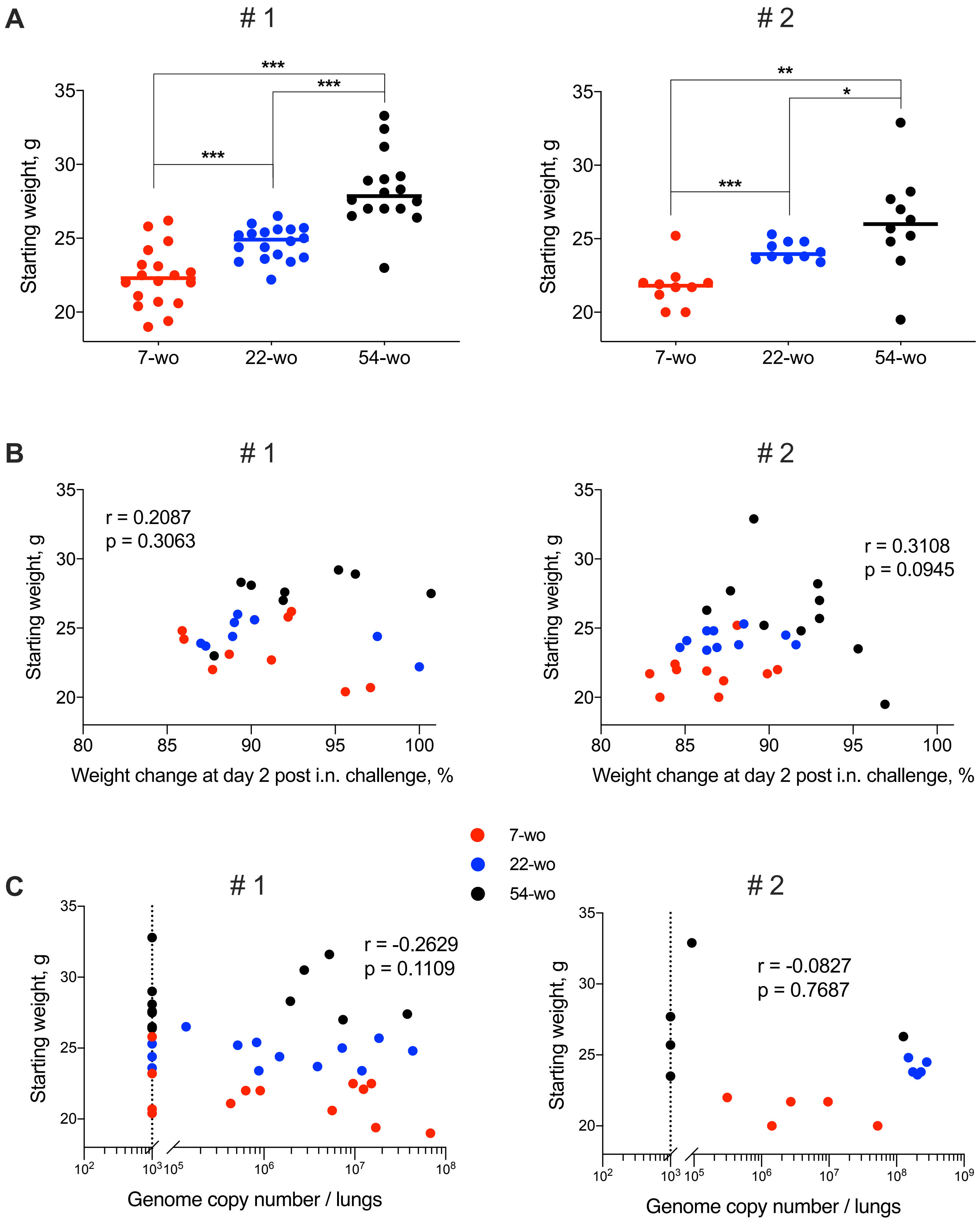

Supplementary figure 9. Starting body weight of the animals does not influence the severity of VACV infection. Groups of 7-, 22- and 54-wo C57BL/6 mice were vaccinated and challenged as described in Fig. 6. Data shown for two experiments: #1 (left panels) and #2 (right panels). (**A**) Starting body weights were measured on the immediately before intranasal challenge. Medians are shown; p values were determined by the Mann-Whitney test, * = p<0.05, **=p<0.01, ***=p<0.001. (**B**) Spearman correlation analysis of starting body weight versus weight change at day 2 post i.n. challenge. (C) Spearman correlation analysis of starting body weight versus VACV load in the lungs measured at 12, 24, or 48 h (for #1) and at 48h only (for #2) after intranasal infection. Dashed lines indicate limit of sensitivity.

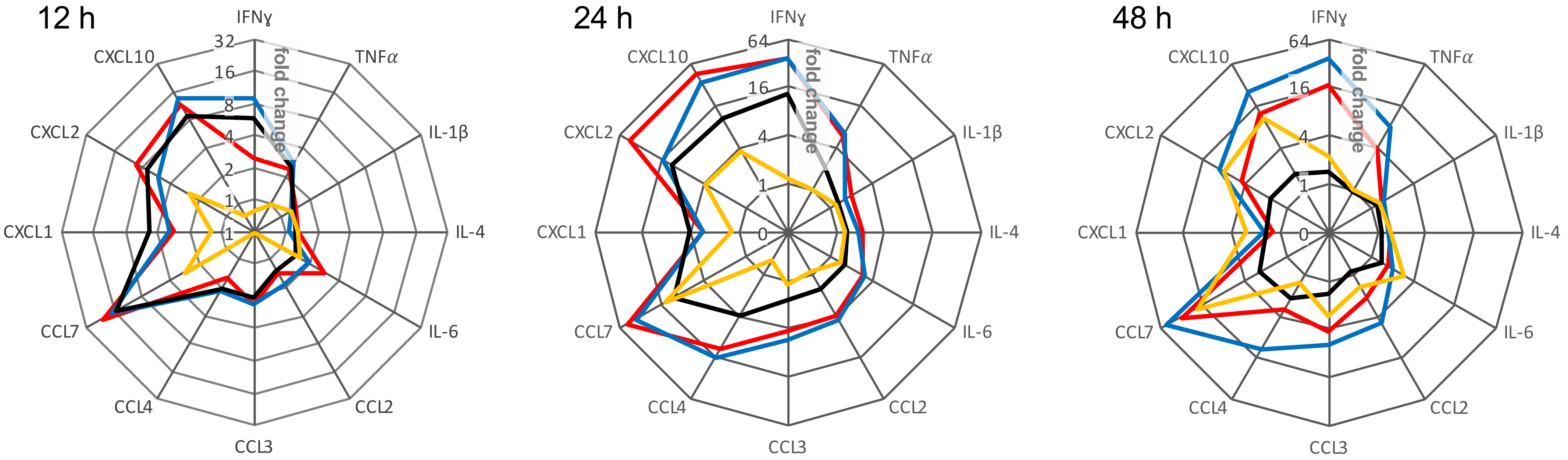

Supplementary figure 10. Alternative representation of data from Fig. 7. Groups (n=4-5) of 7-, 22- or 54-wo C57BL/6 mice were infected intradermally with 10^4 PFU of VACV WR. After 33 d, the mice were infected intranasally with VACV WR and the lungs were collected at 12, 24 and 48 h later. The levels of cytokines and chemokines were measured by multiplex assay (Luminex). The radar plots display data from the three age groups (7-wo in red, 22-wo in blue and 54-wo in black) together with naive (mock-vaccinated) animals (in yellow). Data presented are the fold change from the baseline levels of each cytokine/chemokine in mice before intranasal challenge. Means are shown.

Supplementary table 1. Monoclonal antibodies and dyes used for staining cells prior to analysis by flow cytometry.

Antibody/dye	Clone	Source
B220-PerCP	RA3-6B2	103234, BioLegend
Bcl-6-PE	K112-91	561522, BD Biosciences
CD3-APC	145-2C11	553066, BD Biosciences
CD3-BV421	145-2C11	562600, BD Biosciences
CD4-APC-H7	GK1.5	560181, BD Biosciences
CD4-BUV395	GK1.5	563790, BD Biosciences
CD5-BV421	53-7.3	562739, BD Biosciences
CD8-BB515	53-6.7	564422, BD Biosciences
CD8-BV605	53-6.7	100744, BioLegend
CD11b-PE	M1/70	101208, BioLegend
CD11c-BV650	N418	117339, BioLegend
CD19-BV421	1D3	562701, BD Biosciences
CD44-BB515	IM7	564587, BD Biosciences
CD45-PerCP	30-F11	557235, BD Biosciences
CD62L-APC-C7	MEL-14	104428, BioLegend
CXCR5-BV421	L138D7	145512, BioLegend
Ki-67-FITC	SolA15	11-5698-82, eBioscience
Ly6C-APC	HK1.4	128016, BioLegend
Ly6G-APC-H7	1A8	565369, BD Biosciences
MHC Dextramer H-2Kb/TSYKFESV/PE		JD3267-PE, Immudex
NK1.1-BV421	PK136	562921, BD Biosciences
NK1.1-BV605	PK136	108739, BioLegend
PD-1-APC (J43)	J43	562671, BD Biosciences
Siglec-F-BB515	E50-2440	564514, BD Biosciences
Zombie Green Fixable Viability Kit		423111, BioLegend
Zombie Violet Fixable Viability Kit		423113, BioLegend
PE Mouse IgG1, κ Isotype Control	MOPC-21	554680, BD Biosciences
FITC Rat IgG2a kappa Isotype Control	eBR2a	11-4321-80, eBioscience
